# Challenges in “probing spectroscopic probes” for noninvasive simultaneous disease diagnosis

**DOI:** 10.3389/fchem.2024.1463273

**Published:** 2025-01-08

**Authors:** Lopamudra Roy, Shweta Paul, Amrita Banerjee, Ria Ghosh, Susmita Mondal, Monojit Das, Nivedita Pan, Ishitri Das, Soumendra Singh, Debasish Bhattacharya, Asim Kumar Mallick, Samir Kumar Pal

**Affiliations:** ^1^ Department of Applied Optics and Photonics, University of Calcutta, Kolkata, India; ^2^ Department of Paediatric Medicine, Nil Ratan Sircar Medical College and Hospital, Kolkata, India; ^3^ Department of Physics, Jadavpur University, Kolkata, India; ^4^ Department of Chemical and Biological Sciences, S. N. Bose National Centre for Basic Sciences, Kolkata, India; ^5^ Department of Zoology, Vidyasagar University, Midnapore, India; ^6^ Department of Zoology, Uluberia College, University of Calcutta, Howrah, India; ^7^ Department of Gynecology and Obstetrics, Nil Ratan Sircar Medical College and Hospital, Kolkata, India

**Keywords:** noninvasive, haemoglobin, bilirubin, oxygen saturation, portable, low cost

## Abstract

Noninvasive diagnosis of human diseases relies on the detection of molecular markers (probes) in a painless manner. Although extrinsic and intrinsic molecular markers are often used, intrinsic disease probes (molecular markers) are preferable because they are naturally present in our body, and deviation in their concentration from normal levels clearly indicates anomalies in human bodies, that is, diseases. In this study, we report noninvasive spectroscopic measurements of total haemoglobin (Hb), bilirubin, and the ratio of oxy- and deoxyhaemoglobin as disease markers for anaemia, jaundice, and oxygen deficiency, respectively, using a meticulously designed optical fibre probe. The challenges in designing the fibre probe for simultaneous noninvasive detection, including optical power, spectral density of the probing light, and resolution of the spectrometer, were found to be critical to accurate measurements. Finally, a fibre-less, highly portable, and low-cost prototype was developed and tested in human clinical trials for the diagnosis of diseases, and these results were compared with conventional techniques (blood tests).

## Introduction

The ability to monitor changes in the concentration of intrinsic biomarkers in the skin (particularly oxy- and deoxyhaemoglobin and bilirubin) *in vivo* and in real time is a key component of personalized patient care. For example, changes in the concentrations of haemoglobin (Hb) and bilirubin can cause anaemia and jaundice (An et al., 2021; Roche and Kobos, 2004), which, if not monitored closely, can progress to severe complications. In addition, changes in the levels of oxy- and deoxyhaemoglobin in the blood may cause hypoxic conditions within the physiological system, resulting in adverse impediments. The levels of haemoglobin and bilirubin are interlinked as deviations in one biomarker affect the concentration of the other, thereby influencing the disease conditions. Persistent jaundice causes a significant increase in bilirubin levels, followed by a decrease in haemoglobin levels (Alkhotani et al., 2014), leading to reduced oxygen supply to the liver. This impairs the bilirubin conjugation ability of the liver, simultaneously causing pathological conditions such as jaundice, anaemia, and hypoxia (Bizuneh et al., 2020).

The current gold standard for haemoglobin estimation is the automated haematology analyser, which measures haemoglobin concentration in blood samples in a laboratory setting (Srivastava et al., 2014). Although numerous other methods, such as HemoCue, haemoglobin colour scale, and copper sulphate method, are available, they require a high level of technical skills for interpretation and involve invasive blood sampling techniques (Srivastava et al., 2014). In addition, the contemporary method for the detection of total serum bilirubin concentrations (TSB) involves painful blood sampling (Yap et al., 2002; Meites, 1988), which is associated with multiple long-term consequences like infection and osteomyelitis, especially in neonates (Meites, 1988; Lilien et al., 1976; Lemont and Brady, 2002). In this regard, transcutaneous bilirubinometry (TcB) can become an alternative to repetitive blood sampling and its associated complications. However, certain inherent limitations affect its widespread use in hospital settings, including variations in accuracy across different skin colours and in babies with hyperbilirubinemia associated with risk factors (Maisels, 2015; Maisels, 2012; Bosschaart et al., 2012). On the other hand, the available transcutaneous pulse oximeters are state-of-the art technology for the continuous estimation of oxygen saturation among neonates. Although pulse oximeters have been correlated with blood oxygen saturation and have been used in hospital settings for more than a decade, they have certain limitations, including their vulnerability to the motion of the subject and overestimation of the arterial oxygen saturation (SpO_2_) at less than 90% saturation, which limits their effectiveness in infants suffering from heart diseases (Dawson et al., 2013; Kim et al., 2019). In addition, several articles report optical imaging techniques based on the absorption of light by haemoglobin to indicate normal biological or pathological processes. Optical imaging of biomarkers can be used to measure parameters such as haemoglobin concentration, oxygen saturation, and blood flow (Li et al., 2021). One such technology used orthogonal polarization spectral imaging to provide high-quality digitised images of the microcirculation using reflected light (Nadeau and Groner, 2001). However, its application was limited to laboratory settings and has not been confirmed in adult and paediatric populations, where anaemia is more commonly encountered. A noninvasive approach to Hb estimation based on the image analysis of a specific conjunctival region was also reported by Dimauro et al. (2018), and it was used as a screening device to identify anaemic patients. The analysed RGB attributes of the acquired digital images of palm, soles, and forehead were used as parameters to screen adults and neonates for jaundice (Castro-Ramos et al., 2014). Furthermore, the invasive method of blood sampling for disease diagnosis imposes a huge economic burden on both hospitals and patients (Lyman et al., 2005). According to the Institute for Health Metrics and Evaluation, the Global Burden of Disease (GBD) study conducted in 2013 reported that anaemia affected 27% of the world’s population (1.93 billion people) (Kassebaum and GBD 2013 Anemia Collaborators, 2016). Neonatal jaundice affects more than 60% of term and 80% of preterm newborns during the first week of life (Ebbesen et al., 2002; Keren et al., 2009; Moyer et al., 2000) and requires continuous monitoring of total serum bilirubin levels (TSB). The conventional method of blood sampling to estimate TSB levels is expensive, labour-intensive, time consuming, and dilatory, which prevents the possibility of immediate diagnosis.

To date, the lack of a portable, easily operable, inexpensive, and accurate probe has hindered the widespread adaptation of probing intrinsic human disease markers in public health settings. The corresponding challenges associated with the design of noninvasive probes include accuracy in the measurement and manoeuvrability of the probes by minimally trained personnel in the point-of-care use. In this study, we aim to develop a noninvasive point-of-care probe for the estimation of Hb, TSB, and oxygen saturation for the diagnosis of anaemia, jaundice, and hypoxia screening. Earlier, we developed probes using optical fibres for haemoglobin and bilirubin estimation from the vascular bed of the bulbar conjunctiva (Sarkar et al., 2017; Polley et al., 2015). As the conjunctiva in all humans is transparent and has a white sclera as background, the accuracy and sensitivity of the probe are independent of the skin colour and other pathological conditions of the subjects. The easy access to the conjunctiva and its high vascular visibility ensure high accuracy and sensitivity in the probe. In addition, we also developed a fibre-optic probe associated with a grating-based spectrometer for the measurement of bilirubin levels in newborns (TcB) as an alternative for TSB (Halder et al., 2019; Halder et al., 2020). In another developed fibre-optic probe associated with a grating-based spectrometer, called SAMIRA, we estimated Hb, TSB, and oxygen saturation, which are well-known markers for anaemia, jaundice, and SpO_2_ in neonatal subjects, respectively. The reliability of the collected data, which is an important challenge for noninvasive bio-medical probe design, has also been addressed by incorporating an in-built machine learning algorithm (Banerjee et al., 2023). Although the SAMIRA probe is found to have a good correlation with the invasive blood test, the use of optical fibres limits its usability in point-of-care primary healthcare settings. The use of a grating-based spectrometer is also one of the limitations to design a biomedical probe.

In our attempt to address the above mentioned challenges, first, we investigated a diffuse reflectance probe that replaced the grating-based spectrometer by using several monochromatic LEDs and a photo-transistor as the detector. The developed probe revealed limited accuracy in the diagnosis of anaemia, jaundice, and oxygen saturation (AJO) in neonatal subjects. Second, we studied the role of a grating-based micro-spectrophotometer with a white LED light source to improve the quality of AJO data. In the development, we used a handheld probe containing the micro-spectrophotometer, a white LED source, and associated electronics, which are in communication with a home-made computer *via* Bluetooth. Although the AJO data quality improved, the system was found to have limitations in manoeuvrability for point-of-care applications. Finally, we developed a compact, fibre-less, pocket-sized diffuse reflectance optical probe containing a filter-based spectrometer (customised to detect eight wavelengths in the visible spectrum) and four LEDs (two white and two blue) integrated into a multi-layered PCB with SMD electronic components. The developed probe was found to provide AJO data from the neonatal nail bed, consistent with results of the blood test.

## Methods

### Hardware

All the fibre-optic probes, called SAMIRA, discussed in this study, are based on the principle of diffused reflectance spectroscopy. Details of hardware and associated indigenously developed software for monitoring the spectral response from the conjunctiva and nail bed are discussed in earlier works by Sarkar et al. (2017), Polley et al. (2015), Halder et al. (2019), and Banerjee et al. (2023).

For the development of a diffuse reflectance-based fibre-less probe, named mini-ASAMIR, which replaces the grating-based spectrometer, we used six monochromatic LEDs with emission wavelengths of 365, 460, 520, 560, 620, and 940 nm, respectively. Whereas the diffuse reflected light from neonatal nail beds at wavelengths of 460 and 560 nm reveals bilirubin and haemoglobin, respectively, the ratio of intensities at 620 and 940 nm obtained from the phototransistor depicts oxygen saturation in the subjects (see below). The handheld probe, incorporating the micro-spectrophotometer from Hamamatsu C12880MA Spectrometer, four white light LEDs (3W, 400–700 nm, 700 LUX, 4.78 mW optical power), and associated electronics (Esp32 microcontroller-based), was connected to an indigenously developed mini-computer for the data analysis; this setup was used for noninvasive probing of AJO data from the nail beds of neonatal subjects (see later). We call the probe as ASAMIR to distinguish it from other optical probes. The AJO data were transferred to the mini-computer through a Bluetooth connection for de-convolution of the obtained spectra using our developed graphical user interface on the LabVIEW (National Instruments) platform.

The final development, called the HBO probe, consists of two white SMD LEDs, two blue LEDs, a custom-made filter-based spectrophotometer (consisting of channels at 415, 445, 480, 515, 555, 590, 630, and 680 nm), and an ESP32-based microcontroller (see below). This developed optical probe primarily consists of three distinct parts, namely, the optical detection head, backend electronics, and analysing embedded software. The detection head consists of four SMD LEDs (two white LEDs and two blue LEDs) and a filter-based spectrometer connected to the embedded backend electronics to collect optical signals from the head. The introduction of two blue LEDs with peak maxima at 480 nm is to compensate for the low spectral density of white light in this region, allowing reliable quantification of bilirubin-peaking absorption at 470–480 nm by the device (Guo et al., 2023). The analysing body consists of a microcontroller (powered by the Tensilica Xtensa LX6 Microprocessor), a rechargeable battery unit, and a battery charging circuit. The innovation lies in the development of a separator cap on the detection head, positioned between the neonate’s nail bed and the detector, maintaining a gap of 1.5 mm for the efficient collection of diffused light from the subjects. This spectral response from the detector is transferred to the microcontroller for further processing of the data. Furthermore, the distance between the detector and the light source (4 mm) was maintained such that the penetration depth of the light reached the vascular bed of the neonate’s underneath the nail bed following the principle of the Kubelka–Munk formulation. It is worth noting that the HBO probe is optimised for light intensity to avoid interference from other physical parameters in the neonatal subjects, including nail thickness and its curvature, ensuring accurate probing of blood parameters from the vascular bed underneath the nail bed. This continuous optimisation of light intensity by pulse width modulation (PWM), illumination time, *etc.*, is controlled by this microcontroller. The inbuilt Wi-Fi modules of the microcontroller are responsible for continuous data logging of this portable and IoT-enabled optical probe. The intensity values obtained in these channels, corresponding to 480, 555, and 590 nm of the filter-based detector, were used for the calculation of the instrumentation index of bilirubin, SpO_2_, and haemoglobin, respectively. It is worth mentioning that anaemia, jaundice, and hypoxia in adult subjects cannot be measured using the probe described in the work as the thickness/morphology of the nail bed varies from subject to subject. On the other hand, the nail bed of the neonatal subjects is less prone to inter-subject variation.

### Study design and subjects

The study included a total number of 1,104 neonates with gestational ages ranging from 30 to 40 weeks. The mini-ASAMIR probe, consisting of a customised array of six LEDs and a phototransistor, was calibrated in 150 neonates, and validation was performed in 150 neonates. An additional 904 neonates were recruited for the calibration and validation of the ASAMIR probe. In order to obtain more accurate results in the pocketable version, 200 neonates were recruited for the calibration and validation of the HBO probe. It is worth mentioning that the recruitment of neonates was not consecutive as not all physicians practicing in the department were involved in the study. The neonates receiving treatment under the physicians associated with the study were included. Possible selection bias was avoided following the approach described by Hammer et al. (2009). Random assignment of doctors (a general policy for the public hospitals in India), the large time frame of the study (15 months), a reasonably large sample size, collection of data throughout a 24-h window, and sufficient number of subjects in each subcategory (i.e., stratification of samples) all helped in avoiding sampling bias.

### Haematological measurement

For simultaneous measurement, approximately 2 mL of blood was collected for the conventional TSB and Hb test within 30 min of data collection from the developed probes. The TSB of the subjects was quantitatively determined by the 2,5-dichlorophenyldiazonium tetrafluoroborate (DPD) diazo method described by Garber (1981), using the commercially available test kit (Autospan Liquid Gold, Span Diagnostics, India) within 1 h of blood collection in the Central Laboratory, NRSMH. For the test, serum was first isolated from the collected blood and then examined using the test kit. To prevent the photoreduction of bilirubin, the serum samples were carefully kept in the dark at 4°C before analysis. For haemoglobin, the collected blood samples were subjected to an automated haematology analyser (Sysmex KX-21) (Fares, 2001) for complete blood count (CBC) analysis. All the guidelines provided by the National Accreditation Board for Testing and Calibration Laboratories (NABL) (Kanagasabapathy and Rao, 2005) were followed to maintain the accuracy and precision of the techniques. The coefficient of variance for the hospital laboratory was targeted at <6%. During the study period, each of the actual variance values, assessed every 3 months, ranged from 3% to 5%.

### Ethical considerations

For the present work, all necessary ethical permissions were obtained from the Institutional Medical Ethics Committee, NRSMH, Kolkata (Ref. No. No/NMC/439, dated 27 January 2020). All studies involving human subjects were performed following the Declaration of Helsinki (WMA, 2013) and guidelines provided by the Indian Council for Medical Research (ICMR), Govt. of India. Written informed consent was obtained from parents or legal guardians who agreed to participate in the study after understanding the details of the study and its consequences. All data and information about the subjects were anonymised, kept confidential, and used only for this study.

### Statistical analysis

Analysis of the data was done using descriptive statistical analysis, simple linear regression analysis, and the Bland and Altman method (Bland and Altman, 1986; Bland and Altman, 1994). For the correlation between the values obtained from the developed optical probes and the gold standard, linear regression and the Bland–Altman method were used (Banerjee et al., 2023).

## Results and discussions

**FIGURE 1:**
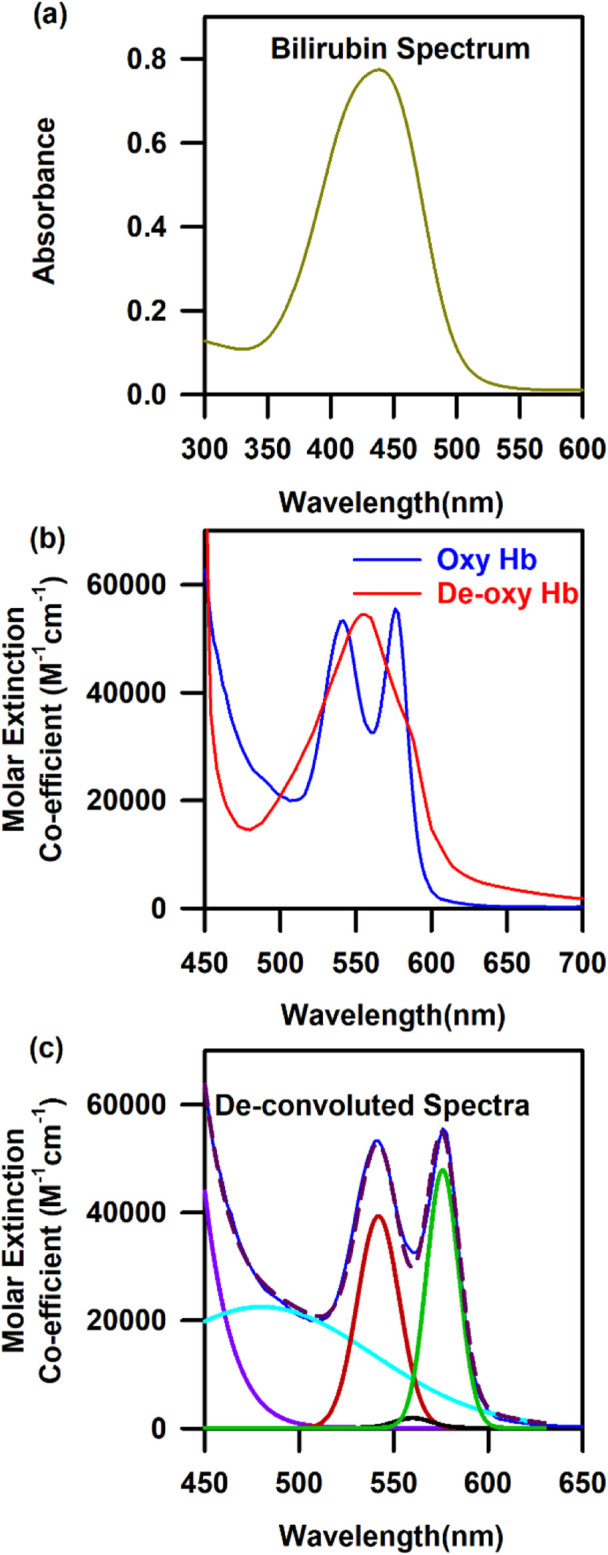
Characteristic spectra of three model probes of the human blood: **(A)** Absorbance spectra of bilirubin. **(B)** Characteristic spectra of oxygenated and de-oxygenated haemoglobin. **(C)** De-convoluted spectra of oxygenated and de-oxygenated haemoglobin into five Gaussian curves with their centre at 462.92, 539.34, 568.09, 577.2, and 620 nm.

**FIGURE 2:**
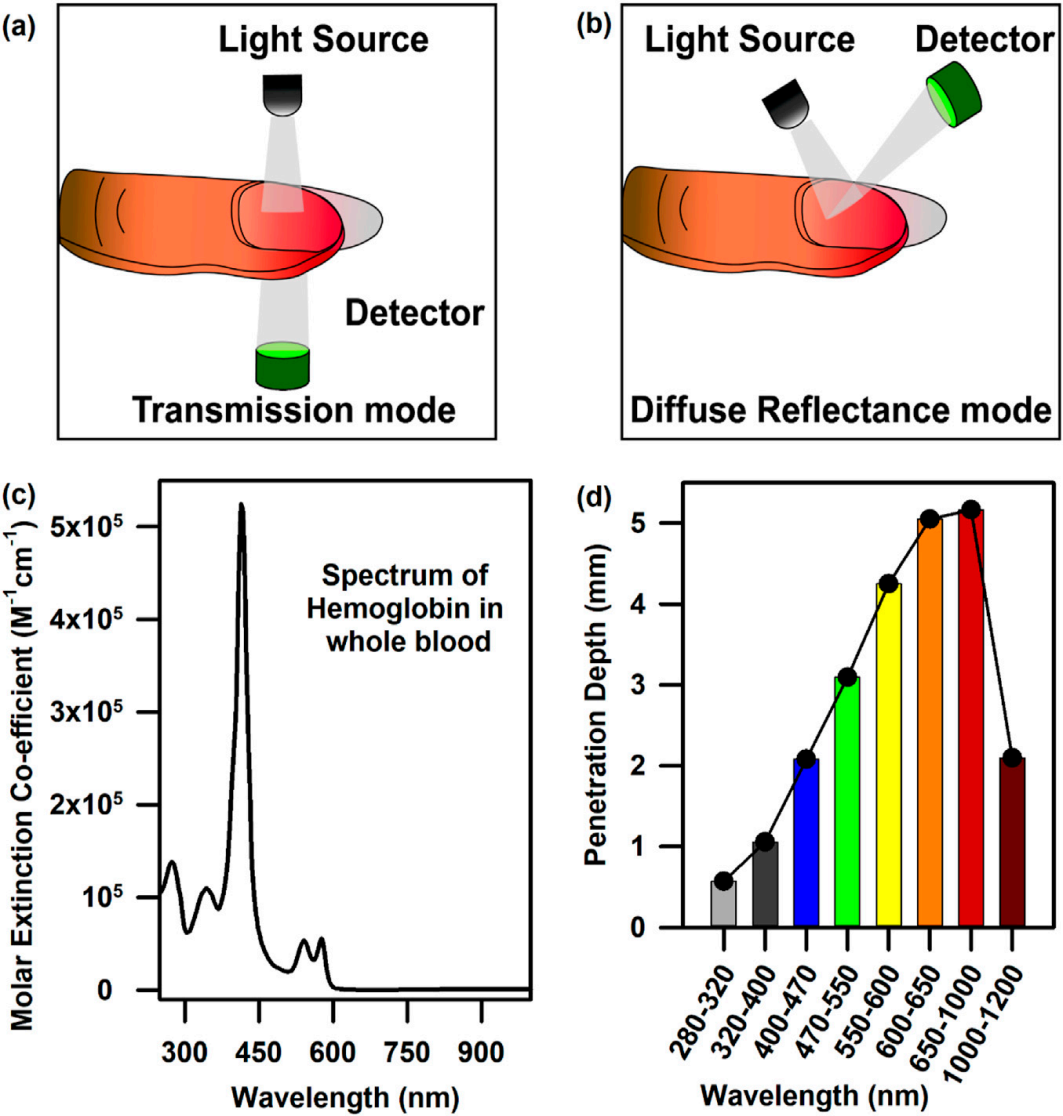
Mode of data collection and light penetration into tissues: **(A)** Data collection from the vascular bed underneath the nail bed in the transmission mode. **(B)** Data collection from the vascular bed underneath the nail bed in the diffuse reflectance mode. **(C)** The full UV-Vis-NIR spectrum of haemoglobin in the whole blood. **(D)** Penetration depth of light as a function of wavelength.

The three of many molecular probes, namely, haemoglobin, bilirubin, and oxygen content, which are omnipresent in our blood, are useful in diagnosing anaemia, jaundice, and hypoxia, respectively (An et al., 2021; Roche and Kobos, 2004). Hence, the design of a suitable probe and associated challenges for the estimation of haemoglobin, bilirubin, and oxygen saturation from blood noninvasively are the main objectives of the current article. Figures 1A, B show the characteristic bilirubin and oxygenated and de-oxygenated haemoglobin absorption spectra in the visible region, respectively. The whole blood spectrum has been de-convoluted to obtain five independent signals at five wavelengths (462.92, 539.34, 568.09, 577.20, and 620.00 nm, Figure 1C). The peak wavelengths are chosen on the basis of the pattern of absorption of oxygenated haemoglobin, de-oxygenated haemoglobin, and bilirubin. The peak wavelength of 462.92 nm corresponds to the absorption of bilirubin, 539.34 and 577.2 nm correspond to the Q-bands of oxy-haemoglobin, and 568.09 nm corresponds to the de-oxygenated peak of haemoglobin. The absorbance at 620 nm has been considered to correct the baseline of the blood spectrum as the molecular absorption of blood is reported to be minimal, and the overall absorption at this wavelength is contributed by scattering of the blood cells/associated tissues. Figures 2A, B present the basic principles of the optical probes, which rely on spectroscopy-based absorbance for probing the spectral response of the blood flowing in the vascular bed using both transmission and diffuse reflectance modes, respectively. In the transmission mode, the fibre-optic source-detector pair is arranged in a 180° geometry (Figure 2A). Although, in the transmission mode, the collected spectral information is more accurate, provided there is good coupling between the fibre optics and the surface, the results may be sensitive to local in homogeneities such as freckles, hair, and localized vasculature, which may alter the individual measurements (Glennie et al., 2015). However, the source/detector geometry can be selected to match a specific range of expected optical properties and minimise the effect of high scattering. This can be achieved through diffuse reflectance spectroscopy (DRS), where the optical properties are determined from the reflectance spectra (Glennie et al., 2015) (Figure 2B). Typically, in DRS, the fibre optic probes are placed at a radial angle from a source fibre, and the measured intensities can be used to determine both the absorption and reduced scattering coefficients (Glennie et al., 2015; Kim and Wilson, 2010). Figure 2C represents the haemoglobin spectra from UV to NIR. It is noteworthy to mention that the characteristic absorption of oxygenated and deoxygenated haemoglobin in the visible wavelength range is significant. The penetration depth of light in living tissues depends on multiple parameters such as wavelength, intensity, polarisation, coherence, and tissue physiology, such as pigmentation, fibrotic structure, hydration, and composition (e.g., hair) (Zhou et al., 2016). Figure 2D shows a comprehensive representation of the penetration depth of light as a function of wavelength. Steady-state DRS measures the wavelength-dependent intensity of light that has entered and scattered back out of a sample (Kim and Wilson, 2010). The observation of lower penetration depth of NIR-II compared to that of NIR-I may be due to the major water absorption peaks in the latter region, which correspond to vibrational modes at ∼900, ∼1,200, ∼1,400, and ∼1900 nm (Younis et al., 2021).

### Diffuse reflectance and photon path length distribution function

**FIGURE 3:**
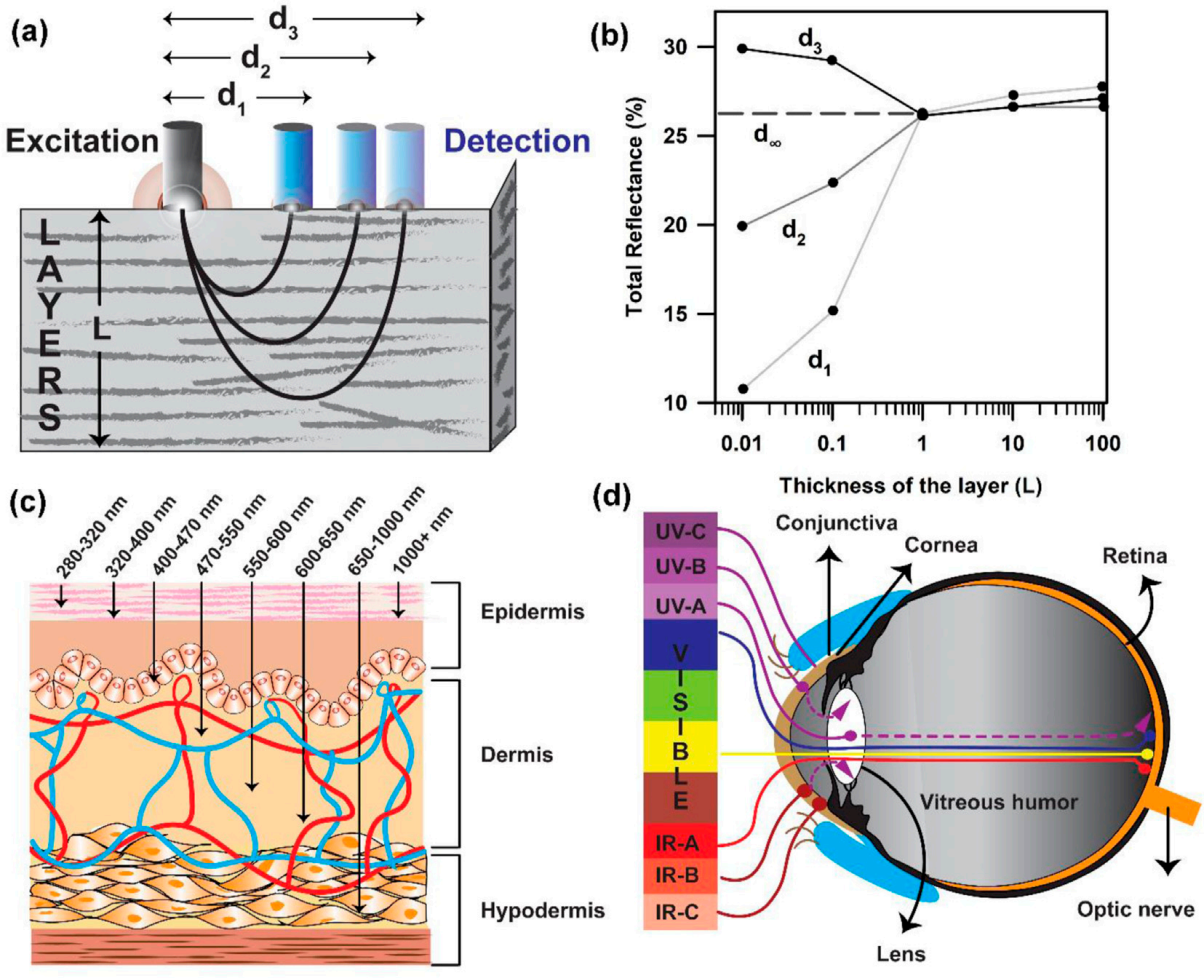
Data collection from the tissue layers following the Kubelka–Munk formulism: **(A)** Probe arrangement with distantly placed fibres for the measurement of diffuse reflectance spectra of tissues. The sampling depth is determined by the light penetration depth (L) and fibre separation distance “d.” **(B)** Total reflectance of light collected as a function of the thickness of the layer. **(C)** Penetration of light of different wavelengths in the skin tissue. **(D)** Penetration depth of light of different wavelengths in the human eye.

The amount of reflected light is dependent on the absorption coefficient µ_a_, which is itself a function of the individual biomarker concentrations (such as oxy- and deoxyhaemoglobin and melanin). The relation between a diffuse reflectance signal and tissue optical properties in terms of photon path length is illustrated in Figures 3A, B. Due to the strong scattering effect by the tissue components, the photon path lengths through the tissue contributing to the measured signal have a distribution (Figure 3A) with a probability function (Stratonnikov and Loschenov, 2001). The intensity of a light signal in the receiving fibre is determined by the absorbing and scattering properties of the tissue and measurement configuration. The path length distribution depends on the scattering tissue properties and the spatial configuration geometry of the fibre probe as its geometry defines the trajectories of photons, which pass through the tissue and reach the detector (Bhattacharyya et al., 2022). As mentioned earlier, the penetration depth of light is dependent on the wavelength of light. Light scattering is inversely proportional to the wavelength (λ), which suggests that higher light penetration depth with less scattering could be achieved at longer wavelengths. For skin, although NIR-I laser light possesses an adequate penetration depth (Figure 3C) (Zhou et al., 2016; Younis et al., 2021; Ruggiero et al., 2016), which is much higher than a visible light photon, the utilisation of NIR laser excitation is yet not the best choice to probe intrinsic biomarkers like haemoglobin due to photodamage (Arkhipov et al., 2016). Prolonged exposure to short-wavelength light (e.g., UVC-A) can seriously harm cells by inducing photochemical reactions (Ruggiero et al., 2016; Francisco et al., 2021). In the eyes, the cornea and conjunctiva absorb strongly at wavelengths shorter than 300 nm (Bearden et al., 2001; Peyrot et al., 2010; Schive et al., 1984). UVC is absorbed in the superficial layers of the cornea, and UVB is absorbed by the cornea and lens. UVA passes through the cornea and is absorbed in the lens (Figure 3D). However, the eyes possess the ability to collect and focus visible radiation in the retina, which is the source of all blood vessels in the eye. Although NIR is also focused by the cornea and lens and transmitted to the retina, it can cause severe thermal damage. On the other hand, water absorption is predominant at wavelengths higher than 1,000 nm, reducing the penetration of light and causing overheating of biological components. Endogenous probes, including haemoglobin and melanin, have a strong absorption of light in the visible spectrum below 600–700 nm (Karpienko et al., 2016; Zonios et al., 2008; Roy et al., 2024). Therefore, an ideal light source should have an absorption peak above 700 nm to allow for deep tissue penetration. Thus, the appropriate light to probe these intrinsic probes is visible light with wavelengths ranging from 400 to 800 nm.

### Probing of haemoglobin and bilirubin from the conjunctiva of the eyes in adult subjects

**FIGURE 4:**
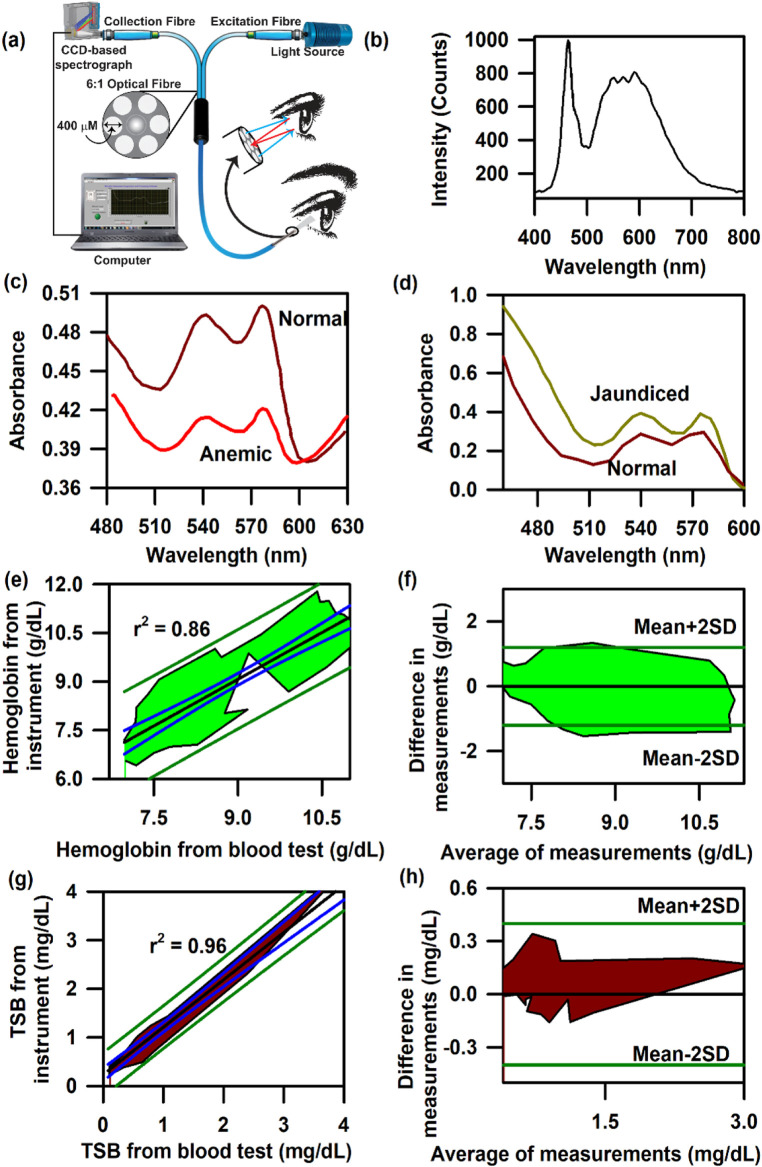
Collection of diffuse reflectance signal from the human conjunctiva to estimate haemoglobin and bilirubin levels in adults: **(A)** Schematic representation of the SAMIRA probe. **(B)** Typical LED spectrum used as a light source in the probe. **(C)** Comparative spectral response of the conjunctiva of a normal volunteer and an anaemic patient. **(D)** The comparative spectral response of the conjunctiva of a normal volunteer and a jaundice patient has been represented. **(E)** Contour of the linear regression analysis of the haemoglobin level from the blood test and that from SAMIRA. **(F)** Contour of the Bland–Altman analysis of the haemoglobin level from the blood test and that from SAMIRA. **(G)** Contour of the linear regression analysis of the TSB level from the blood test and that from SAMIRA. **(H)** Contour of the Bland–Altman analysis of the TSB level from the blood test and that from SAMIRA. The shaded area represents the residence of the data points.

Figure 4A presents the schematic of the SAMIRA probe for probing the diffused reflectance spectral response of the blood flowing in the vascular bed of the bulbar conjunctiva of human subjects (Sarkar et al., 2017; Polley et al., 2015). The typical spectrum of the LED used is depicted in Figure 4B. Figures 4C, D represent the collected comparative spectral response of a normal volunteer and anaemic (Hb = 8.7 g∕dL) and jaundiced patients, respectively. A distinct difference in their spectral appearance is visible; the collected information on the haemoglobin and bilirubin deposited in the conjunctiva of the subjects is represented in the spectral response. We chose the conjunctiva of human eyes as the target organ to estimate the haemoglobin and bilirubin concentration as it is easily accessible, hosts well-oxygenated blood containing a high-density vascular bed (MacKenzie et al., 2016), and has high vascular visibility with a white background in all human subjects. In addition, the noncontact nature of our method ensures no change in tissue morphology, shape, or blood content in the target area. Figures 4E, F present the contour of the linear regression analysis and the Bland–Altman analysis for the samples in which the haemoglobin level exceeds 11 g∕dL (*p*-value < 0.0001, slope = 0.983, and intercept = 0.349). The details of data collection and validation of the probe have been discussed in a previous publication by Sarkar et al. (2017). Similarly, the correlation between the TSB values obtained from the blood serum using the conventional blood test and the probe from the conjunctiva has been illustrated in Figures 4G, H. The contour of the linear regression between the two methods reveals a correlation coefficient, r = 0.99 (*p* < 0.0001; slope 1.026; and *y*-intercept 0.018, Figure 4G). The contour of the Bland–Altman analysis reveals that the maximum deviation of the data points from the mean (mean ± 2 SD) ranges between 0.3 mg/dL. The details of the validation of the probe for the collection of TSB have been discussed in our previous publication (Polley et al., 2015).

### Noninvasive probing of bilirubin from the nail bed of neonates

**FIGURE 5:**
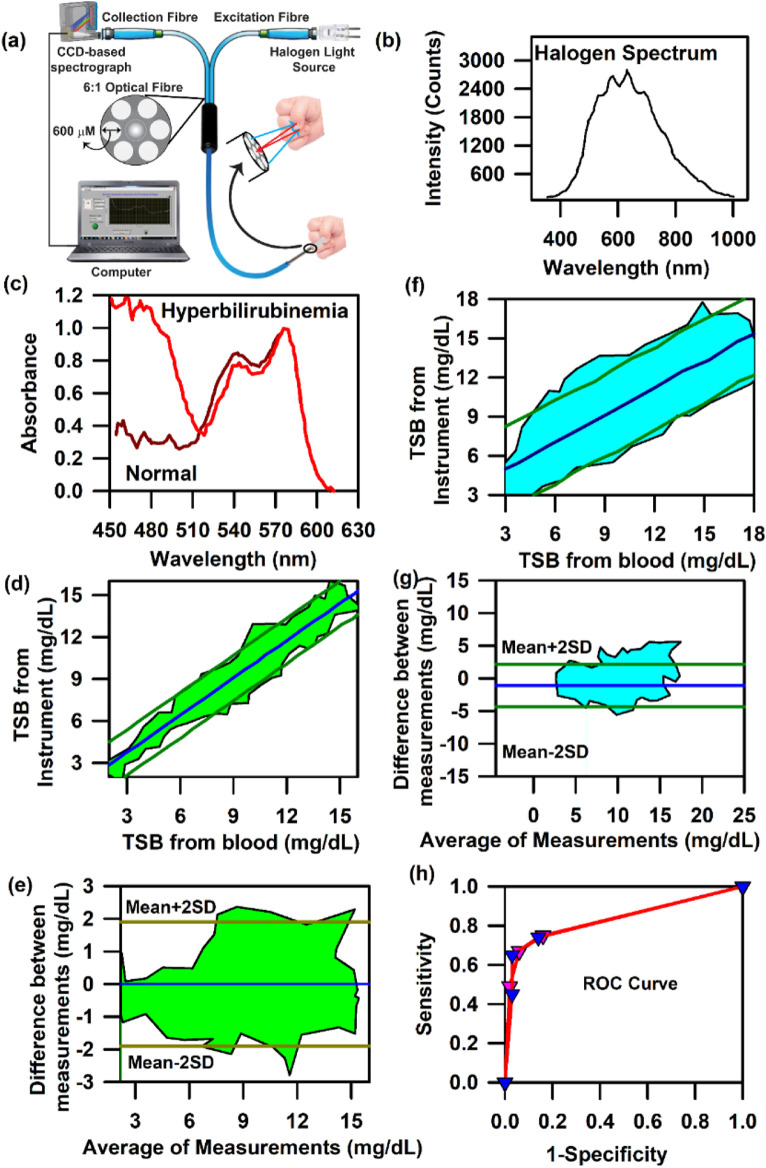
Collection of diffuse reflectance signal from the nail bed of neonates to estimate bilirubin levels: **(A)** Schematic illustration used by the noninvasive, modified SAMIRA probe to collect information on bilirubin from the neonatal nail bed. **(B)** Typical halogen spectra of the light source used in the probe. **(C)** Representative spectra from the probe from a healthy and a jaundiced neonate. **(D)** Contour of the linear regression analysis of the TSB level from the blood test and that from the probe in neonates. **(E)** Contour of the Bland–Altman analysis of the TSB level from the blood test and that from the probe in neonates. **(F)** Contour of the linear regression analysis of the TSB level from the blood test and that from the probe in neonates suffering from hyperbilirubinemia associated with risk factors. **(G)** Contour of the Bland–Altman analysis of the TSB level from the blood test and that from the probe in neonates suffering from hyperbilirubinemia associated with risk factors. The shaded region signifies the residence of the data points. **(H)** ROC curves of the device depicting the accuracy of the probe.

After a clear correlation between the measured transcutaneous bilirubin (TcB) and haemoglobin from the conjunctival vascular bed of adult subjects with the blood test, the need for improvement of the bilirubin detection protocol in newborns was recognized shortly because of the difficulty of accessing the conjunctiva in neonatal subjects. Thus, we have modified the standard SAMIRA probe to one which is more suitable for illuminating the sublingual arcade below the nail plate of the neonate and to send the collected reflectance light (from 350 nm to 800 nm) to the spectrometer (Figure 5A). The whole spectrum of the halogen light source is depicted in Figure 5B. A comparative spectral response between a normal and a jaundice-affected subject is represented in Figure 5C. A clear difference in the spectral patterns is evident, particularly at approximately 470 nm, which is the peak of bilirubin absorption at physiological conditions. Figures 5D, E represent the contour of the linear regression and the Bland–Altman analysis for neonatal subjects with jaundice, respectively. The correlation coefficient, r, was found to be 0.95 and *p* < 0.001. The limit of agreement is ±1.78 mg/dL, with a negative bias of −0.01 mg/dL. The negative bias indicates the predominant tendency of the probe to overestimate the bilirubin levels by 0.01 mg/dL, which is within the standard deviation of the device. The probe was also evaluated in neonates suffering from hyperbilirubinemia associated with risk factors to test its robustness. The contour for the linear regression on neonates with hyperbilirubinemia associated with risk factors revealed a correlation coefficient, r, value of 0.78 (slope 0.64 and intercept 0.23, Figure 5F). The contour for the Bland–Altman analysis on the same subjects reveals a small positive bias of 0.23 mg/dL along with a standard deviation of 1.89 mg/dL (Figure 5G). It is noteworthy to mention that none of the associated risk factors of hyperbilirubinemia, namely, ABO and Rh incompatibility, sepsis, birth asphyxia, or preterm (American Academy of Pediatrics Subcommittee on Hyperbilirubinemia, 2004; Kemper et al., 2022), was found to be a confounding factor. The variability in the accuracy of the measurement of TcB from the nail bed of the neonates was found to be independent of these factors. The area under the curve (AUC) for the receiver operator curve (ROC) was found to be 0.83 (Figure 5H), and the collected data (right or left nail bed) had insignificant differences in the AUC. A comprehensive and detailed analysis has been published in previous studies by Halder et al. (2019) and Halder et al. (2020).

### Noninvasive simultaneous probing of haemoglobin, bilirubin, and oxygen saturation from the nail bed of neonates

One of the major reasons of pathologic hyperbilirubinemia is the excessive production of bilirubin, which is a by-product of haemoglobin breakdown, and the impaired ability of the newborn to excrete it (Dennery et al., 2001). All infants suffering from persistent jaundice have significantly decreased haemoglobin levels and elevated bilirubin concentration in the blood due to the increased bilirubin production from haemolysis, resulting in the simultaneous pathologic conditions of jaundice and anaemia in neonates (Alkhotani et al., 2014; Dennery et al., 2001). It has also been reported that the occurrence of neonatal jaundice is more likely in neonates suffering from birth asphyxia than in neonates without birth asphyxia (Bizuneh et al., 2020; Devi and Vijaykumar, 2017; Omekwe et al., 2014; Kolawole et al., 2016) due to the lack of oxygen supply to the liver, which causes hypoxic damage followed by the impairment of bilirubin conjugation ability of the liver, ultimately resulting in jaundice. Thus, the simultaneous measurement of Hb, TSB, and oxygen saturation (SpO_2_) in neonates is mandatory to manage hyperbilirubinemia.

**FIGURE 6:**
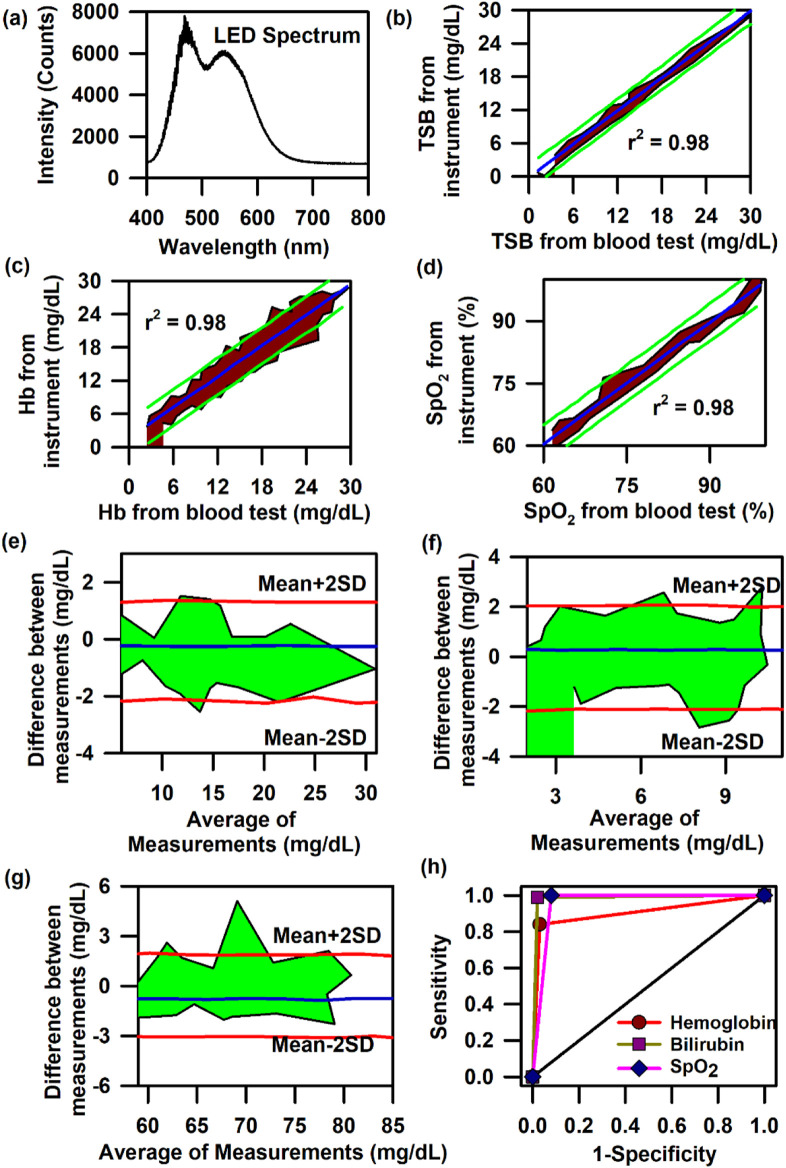
Collection of diffuse reflectance signal from the nail bed of neonates to estimate haemoglobin, bilirubin, and SpO_2_ levels simultaneously: **(A)** Typical LED spectrum of the light source used in the modified SAMIRA probe. **(B)** Contour of the linear regression analysis of the TSB level from the blood test and that from the probe in neonates. **(C)** Contour of the linear regression analysis of the Hb level from the blood test and that from the probe in neonates. **(D)** Contour of the linear regression analysis of the SpO_2_ level from the blood test and that from the probe in neonates. **(E)** Contour of the Bland–Altman analysis of the Hb level from blood test and that from the probe in neonates. **(F)** Contour of the Bland–Altman analysis of the TSB level from blood test and that from the probe in neonates. **(G)** Contour of the Bland–Altman analysis of the SpO_2_ level from the pulse oximetry and that from the probe in neonates. **(H)** ROC curves of the probe depicting the accuracy of the probe for the estimation of Hb, TSB, and SpO_2_ in neonates.

In order to achieve the simultaneous measurement of these three parameters (Hb, TSB, and SpO_2_) in neonates, we have used SAMIRA duly modified for probing neonatal nail beds. However, to decrease the heating effect produced by the halogen lamp source and to increase the shelf life of the light source, we further changed the light source to an LED source. The corresponding spectral response of the LED light source is presented in Figure 6A. The LED source (3W, 400–700 nm, 700 LUX, and 4.78 mW optical power) chosen for this study did not have an attenuated light intensity at ∼460 nm, which is prevalent in commercial LEDs. This LED light source had a significant spectral intensity at ∼460 nm, which was utilised for bilirubin quantification. The contour for the linear regression analysis for TcB quantification revealed r = 0.98, slope = 1.00, and intercept = 0.37; for Hb estimation, r = 0.98, slope = 0.95, and intercept = 0.66; and for the estimation of oxygen saturation, r = 0.98, slope = 0.97, and intercept = 1.77 (Figures 6B–D). The contour for the Bland–Altman analysis for the estimation of haemoglobin and bilirubin reveals a mean±2SD value of 2 g/dL and 2 mg/dL, respectively (Figures 6E, F). The contour for the Bland–Altman analysis for the estimation of SpO_2_ reveals a mean±2SD value of 3%, which is well within the limits of the standard error (Figure 6G). The AUC of the ROC curve for the estimation of Hb, TcB, and SpO_2_ is 0.9 for all three parameters, indicating a significant correlation between the results obtained from the blood test and the values obtained from the developed optical probe (Figure 6H). The details of the measurement protocol, along with the statistical parameters, are elaborated in a previously published article by Banerjee et al. (2023).

### Development of the mini-ASAMIR and ASAMIR optical probe

**FIGURE 7:**
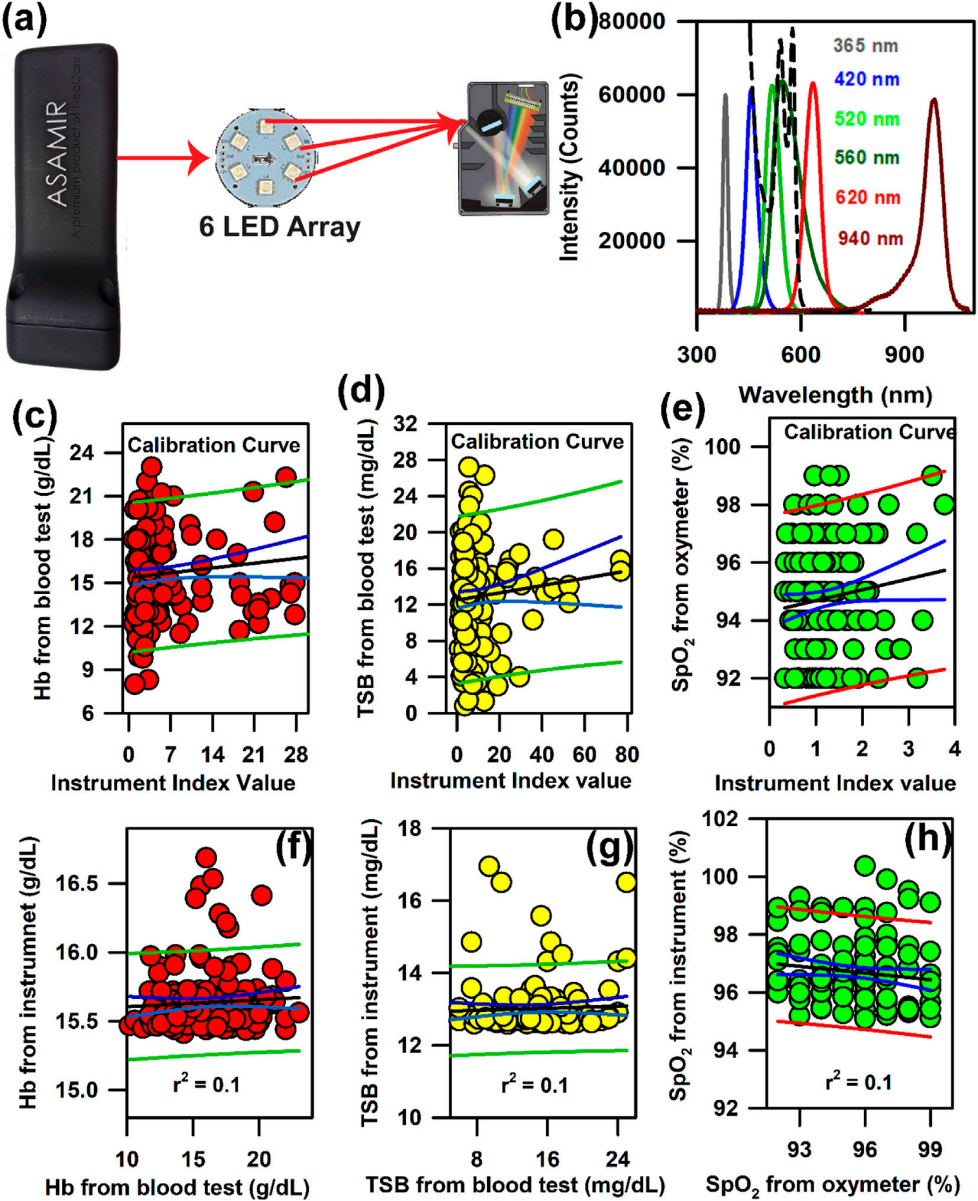
Development of mini-ASAMIR for the collection of diffuse reflectance signals from the nail bed of neonates to estimate haemoglobin, bilirubin, and SpO_2_ levels simultaneously: **(A)** Schematic diagram of the mini-ASAMIR probe with a LED array and a phototransistor. **(B)** Representative LED spectra of the six LEDs in the LED array. A spectrum of whole blood is superimposed in the panel (dotted line) in order to show the location of the interrogation by the respective LEDs for the diagnosis of diseases (see text). Linear regression of the **(C)** haemoglobin level, **(D)** TSB level, and **(E)** SpO_2_ level from the blood test and the instrument index value from the probe for calibration. Linear regression of the **(F)** haemoglobin level, **(G)** TSB level, and **(H)** SpO_2_ level from the blood test and the instrument from the probe for the validation of the probe.

In order to increase the manoeuvrability and to make it portable, we modified the probe in such a way that the light source, detector, and analysis algorithm are incorporated within one compact unit. The schematic of mini-ASAMIR is shown in Figure 7A. The spectral responses of the LED light sources with their peak maxima are shown in Figure 7B. After the collection of photons carrying information from the tissue surface, the in-built algorithm calculated the following instrument index function. For the calibration of Hb and TSB, we followed the principles of absorption spectrometry, that is, log *I*_*0*_ */I* is equal to the absorbance of the analyte to be measured, where *I*_*0*_ is the intensity of the LED light source and I is the diffused reflected intensity. As *I*_*0*_ is the constant factor for all measurements and *I* changes with the change in the concentration of the analytes (Hb and TSB), the instrumentation index was calculated as a reciprocal of the diffused reflectance intensity (*I*). For the estimation of Hb and TcB, the reciprocal of the intensity values of 520 nm and 460 nm was calibrated using the obtained Hb and TSB values of the blood test, respectively. The instrumentation index for the estimation of SpO_2_ was calculated using the ratio of the intensity values at 620 nm and 940 nm (Mannheimer et al., 1997). The linear regression analysis for the calibration of the probe for the estimation of the three blood parameters is shown in Figures 7C–E, respectively. However, validation of the mini-ASAMIR on 150 neonates for the three blood parameters did not yield good correlation between the values obtained from the blood test and those obtained from the probe. The correlation coefficient, r, was 0.1 for all three blood parameters (Figures 7F–H). The relatively poor performance of the probe was probably due to the broad spectral width of the LED sources that caused unwanted absorption of the intrinsic probes during the time of detection and the low optical resolution of the photo detector accompanied by the noise of the photo detector.

**FIGURE 8:**
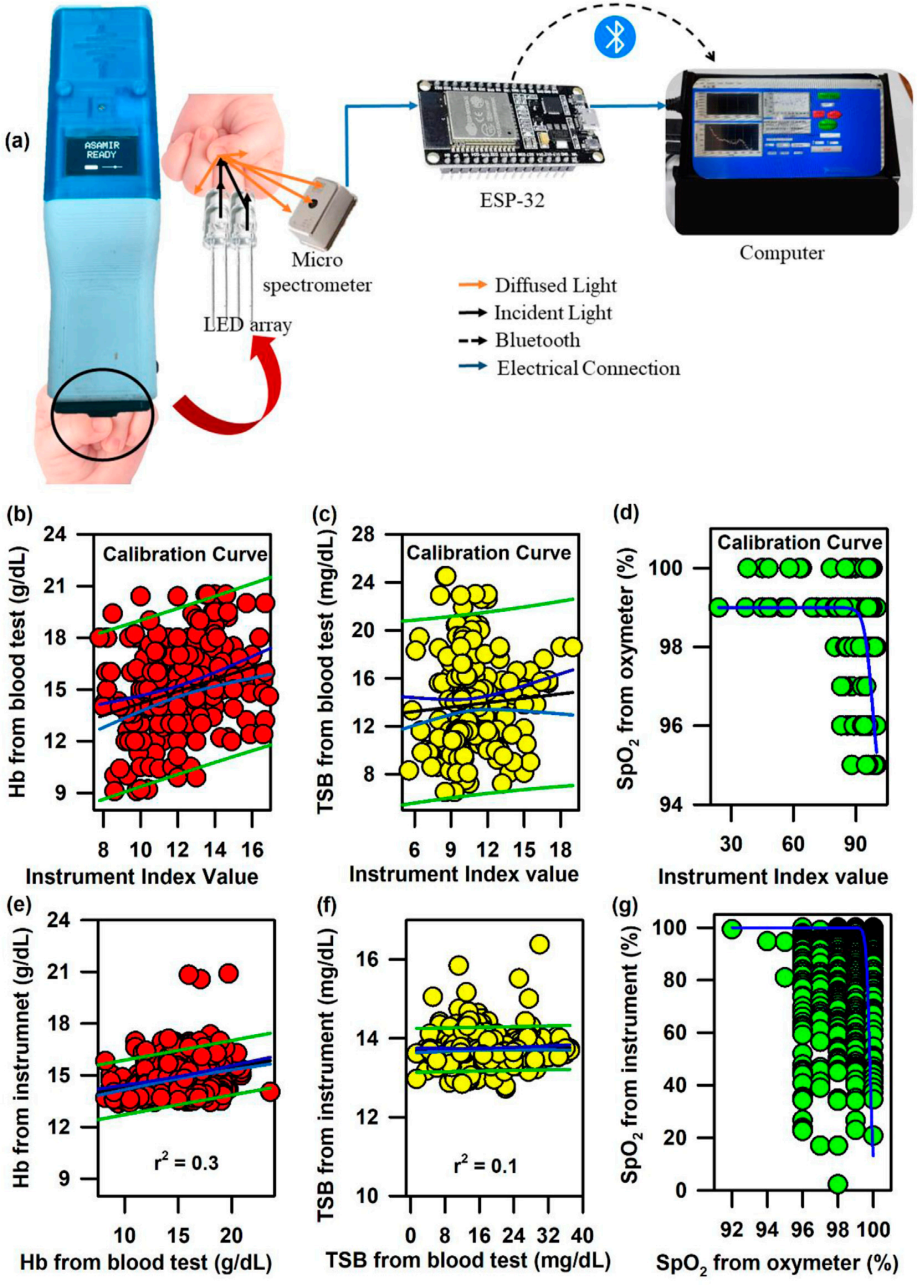
Development of ASAMIR probe for the collection of diffuse reflectance signals from the nail bed of neonates to estimate haemoglobin, bilirubin, and SpO_2_ levels simultaneously: **(A)** Schematic diagram of the ASAMIR probe with a LED array and a micro-spectrophotometer. Linear regression of the **(B)** haemoglobin level, **(C)** TSB level, and **(D)** SpO_2_ level from the blood test and the instrument index value from the probe for the calibration of the device with a new light source and a grating-based micro-spectrophotometer (see text for details). Linear regression of the **(E)** haemoglobin level, **(F)** TSB level, and **(G)** SpO_2_ level from the blood test and the instrument index value from the probe for the validation of the probe with a new light source and a grating-based micro-spectrophotometer (see text for details).

In order to address the challenge, we have developed the ASAMIR probe as described above. The schematic of the ASAMIR probe is shown in Figure 8A. The linear regression analysis for the calibration of the probe on 240 neonates is shown in Figures 8B–D. The validation of the probe on 665 neonates shows a correlation with respect to the values obtained from blood samples (Figures 8E–G), which is better than that of mini-ASAMIR; however, it was relatively lower than that of the SAMIRA probe.

**FIGURE 9:**
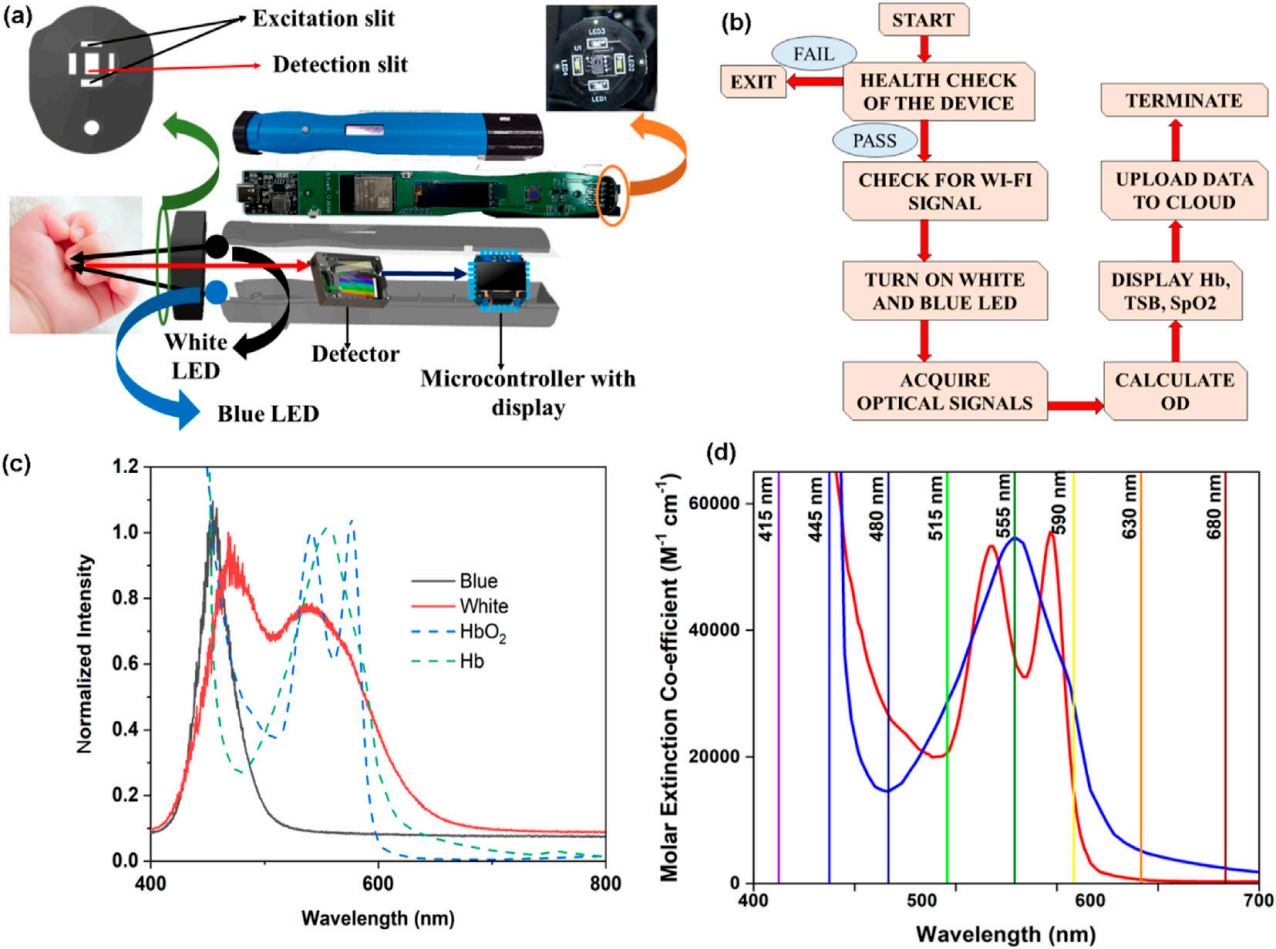
Collection of diffuse reflectance signals from the nail bed of neonates to estimate haemoglobin, bilirubin, and SpO_2_ levels simultaneously from the HBO probe: **(A)** Schematic representation of the HBO probe and the method of data collection. **(B)** Work flow of the HBO probe in real-world use. **(C)** Source spectrum of the white and blue LEDs used in the probe. **(D)** Illustrative representation of the channels of the filter-based spectrometer used in the HBO probe.

**FIGURE 10:**
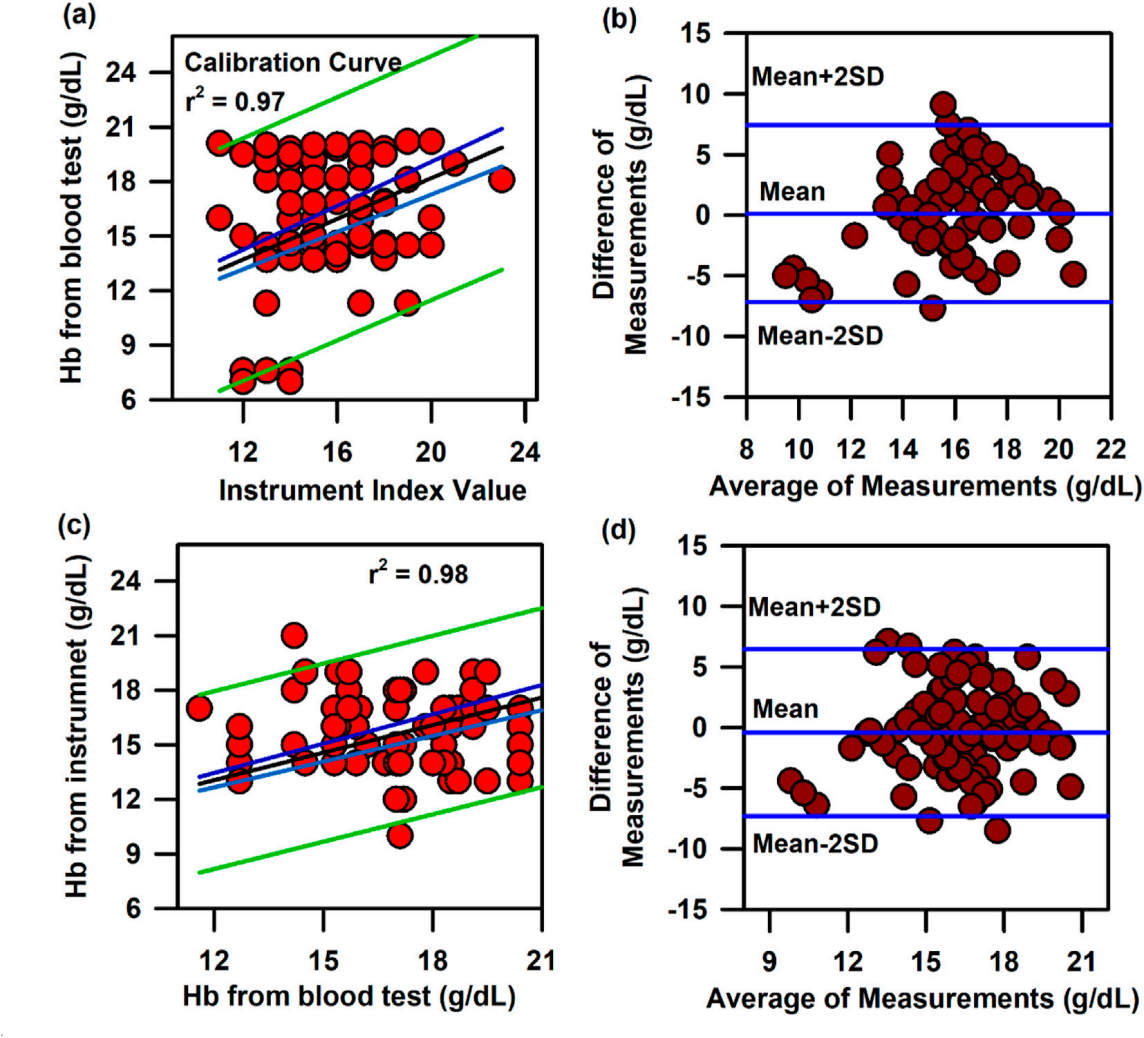
Calibration and validation curve of Hb using the HBO probe: **(A)** Linear regression of the haemoglobin level from the blood test and the instrument index value from the HBO probe with 95% CI and 95% prediction intervals. **(B)** Bland–Altman analysis of the haemoglobin level from the blood test and the instrument index value from the HBO probe. **(C)** Linear regression of the haemoglobin level from the blood test and the haemoglobin level from the HBO probe with 95% CI and 95% prediction intervals for the validation of the HBO probe. **(D)** Bland–Altman analysis of the haemoglobin level from the blood test and that from the HBO probe with mean±2SD.

**FIGURE 11:**
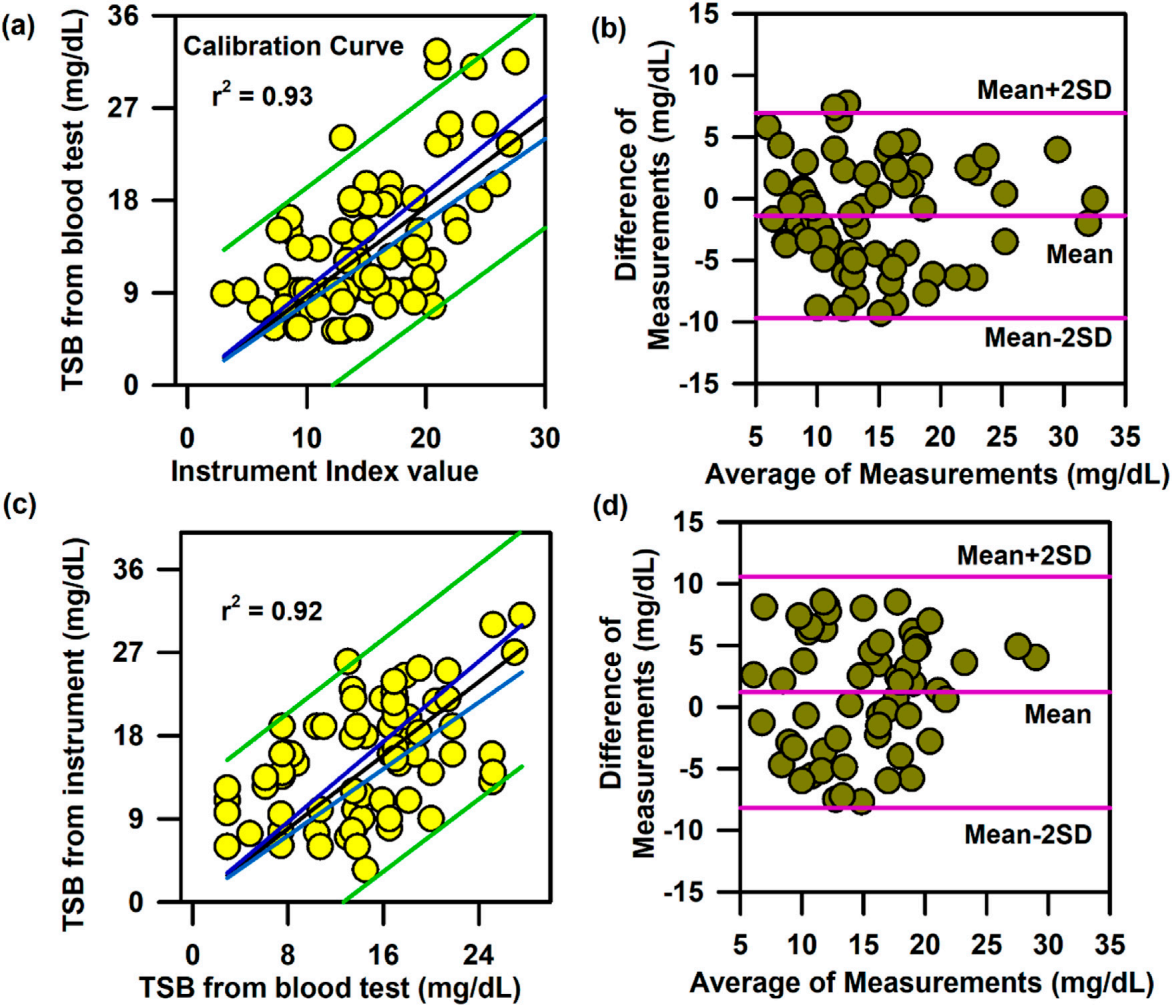
Calibration and validation curve of TSB using the HBO probe:. **(A)** Linear regression of the TSB level from the blood test and the instrument index value from the HBO probe with 95% CI and 95% prediction intervals. **(B)** Bland–Altman analysis of the haemoglobin level from the blood test and the instrument index value from the HBO probe. **(C)** Linear regression of the TSB level from the blood test and the TSB level from the HBO probe with 95% CI and 95% prediction intervals for the validation of the probe. **(D)** Bland–Altman analysis of the TSB level from the blood test and that from the HBO probe with mean±2SD.

**FIGURE 12:**
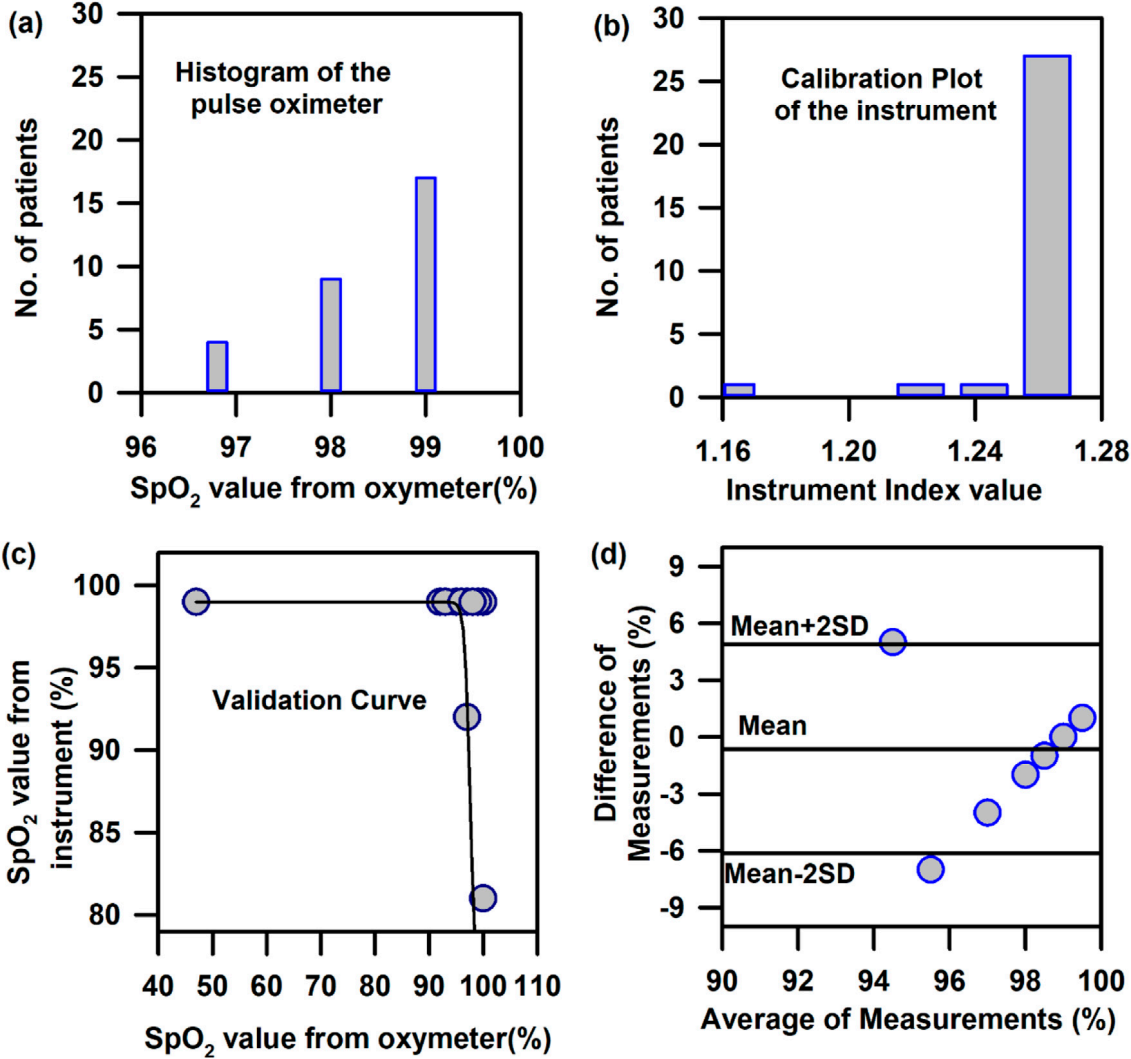
Calibration and validation curve of SpO_2_ using the HBO probe: **(A)** Number of patients corresponding to the instrumentation index value obtained from the instrument for the calibration of SpO_2_. **(B)** Number of patients corresponding to the SpO_2_ values obtained from pulse oximetry for the calibration of SpO_2_. **(C)** Validation of the HBO probe with pulse oximeter values obtained from standard pulse oximeters (Nellcor) **(D)** Bland–Altman analysis of the SpO_2_ values obtained from the HBO probe with pulse oximeter values obtained from standard pulse oximeters (Nellcor).

In the above context, it is extremely important to develop an optical probe for noninvasive detection of AJO parameters, which would be consistent with that of the blood test. The HBO optical probe (Figure 9A), as described above, addresses most of the challenges mentioned earlier, and its mass production is economically viable. The working flowchart of the HBO probe is shown in Figure 9B. Figure 9C represents the spectrum of the excitation source from the SMD LEDs. The different wavelength bands (channels) of the custom-made detector are depicted in Figure 9D, which are distributed across the blood spectrum. The correlation plot of the instrument with the three blood parameters calculated from the standard biochemical test on 100 neonatal subjects shows a linear pattern of dependency. The instrument index values maintain a linear relationship with the values obtained from blood tests for all the parameters. The Pearson’s correlation coefficient, r, was found to be 0.97 (slope = 0.5 and intercept = 7.0) for Hb estimation (Figure 10A), and for the estimation of TSB, the correlation coefficient, r, was found to be 0.93 (slope = 0.8 and intercept = 0.04)(Figure 11A). The Bland–Altman analysis revealed standard deviation values of 3.7 gm/dL and 4.2 mg/dL for the estimation of Hb and TSB, which is well within the standard error (Figures 10B, 11B). The values obtained from conventional pulse oximetry are illustrated in Figure 12A, and the instrumentation index for the estimation of SpO_2_ is illustrated in Figure 12B. It has to be noted that the individual SpO_2_ values of the subjects are well corroborated with the instrumentation index of the HBO probe.

The HBO probe was validated in 100 neonates. Linear regression analysis between the obtained Hb values from the probe and blood tests shows better correlation, with r = 0.98 (slope = 0.5 and intercept = 7.2; Figure 10C). The Bland–Altman analysis revealed a standard deviation value of 3.1 gm/dL (Figure 10D). Linear regression analysis for the validation of TSB values obtained from the blood test and the instrument reveals a correlation coefficient of 0.92 (slope = 0.9 and intercept = 0.01; Figure 11C), and the Bland–Altman analysis shows an SD of ±4.7 mg/dL (Figure 11D). The validation plots for the estimation of SpO_2_ (Figures 12C, D) also show good correlation between the values obtained from the HBO probe and those obtained from the pulse oximeters.

## Conclusion

Our results suggest that noninvasive probing of intrinsic probes of our body (haemoglobin, bilirubin, and oxygen content) is very important for diagnosing anaemia, jaundice, and hypoxia levels in the blood as it helps reduce blood loss and related complications. However, several challenges are associated with the development of an optical probe of accurate diagnosis in the point-of-care setting, particularly in low-to-middle-income-group countries. In our series of optical probe development efforts, relying on a noninvasive spectroscopic strategy based on the principle of diffuse reflectance, which is independent of race, age, and sex of the subjects, we have addressed these challenges. In our latest development of a fibre-less, noninvasive, portable optical probe, we have ensured that biomedical data collection comes from the vascular bed underneath the neonatal nail bed. The distance between the light sources and the filter-based micro-spectrophotometer has been optimised to ensure that the light penetrates to the vascular bed and probes the blood parameters, overcoming the limitations of the conventional TcB and haemoglobin metres. Moreover, the probe could also measure TSB >20 mg/dL, which was achieved by the modification in the illumination intensity and introduction of a blue LED in the source (Guo et al., 2021). In future, the probe would be useful for initial screening and routine examinations to monitor the prognosis of diseases related to anaemia, jaundice, and oxygen saturation.

## Data Availability

The original contributions presented in the study are included in the article/supplementary material; further inquiries can be directed to the corresponding author.
